# Kansas State Veterinary Medical Association

**Published:** 1893-10

**Authors:** N. S. Mayo

**Affiliations:** Sec.


*Kansas State Agricultural College.*—The Kansas Veterinary Medical Association met at Manhattan, March 9th, 1893. The afternoon was spent visiting the State Agricultural College.

The meeting was called to order by President Pritchard. The minutes of the previous meeting were read and approved. Drs. Young and Le May signed the Constitution. Communications were read from Drs. Hunter, Wattles and Nott expressing regrets at being unable to attend the meetings.

Dr. Wattles reported an interesting case of an "equine hermaphrodite." Dr. Wattles also extended an invitation to attend the commencement of the Kansas City Veterinary College.

Dr. Le May gave an interesting talk on the treatment of gunshot wounds in horses, having had considerable experience while at Pine Ridge with his regiment (7th cavalry). He found the most successful method was to treat the wound anticeptically locally, and wait until the ball became encysted or set up suppuration, when it could be removed. He related some very interesting cases.

Dr. Welch, of Salina, reported an interesting case of artificial impregnation of a mare. The semen was caught in a receptacle, mixed with warm milk and by means of a small rubber tube and funnel, was forced into the uterus. The mare was impregnated.

The treatment of herniæ was also discussed thoroughly. Neurotomy was also discussed. The general expression was that the low operation, performed standing, was usually successful, no bad results following. Tenotomy, bulging sole, Demps, pads and rubber-tired shoes were also discussed.

Under the head of unfinished business, the fee bill was brought up, amended and adopted as a general guide to equalize charges throughout the State and for the general information of the practitioner and his client.

The amended fee bill was ordered printed for the use of the members. Dr. L. Orr's resignation as a member of the association was taken from table and accepted.

Under new business a committee, consisting of Drs. Welch, Mayo and Brady was appointed to revise the Constitution and By-Laws and present them at the next meeting.

The association then adjourned to meet at Topeka the Thursday evening of State Fair week in October, 1893.

N. S. MAYO, *Sec.*

